# Points of Interest in the Care of Children’s Teeth

**Published:** 1896-12

**Authors:** 


### Points of Interest in the Care of Children's Teeth.

[*Continued from November Number, page* 548.]

DISCUSSION.

Dr. Geo. L. Field: There is probably no more important duty the dentist is called upon to perform, or one more perplexing and trying, than that of caring for the teeth of children. Here it is that we are first called upon to meet and combat an enemy to peace and happiness of future years, whose chief and strongest ally is the little one brought to us for our professional care and assistance, to meet the approaching aches and pains of a not far-distant future. Coming to us, as they do, with trembling footsteps and cheeks bedewed with tears; looked upon by them, as we are, as the very personification of all that is evil and to be avoided, the question arises: What are we to do, and how are we to do it? Heroic measures certainly will not be most conducive to the end in view, and yet a certain amount of firmness is absolutely necessary, and more or less pain must accompany our efforts or we shall make a most ignominious failure to attain the end sought. We wish to impress upon the mind of our little patient the fact that we are in an effort to do that which shall be of not only temporary, but of future benefit; that we wish to be held in the light of a friend and not of an enemy. But the pain that we necessarily inflict seems to dissipate all hope in our favor. Early impressions are hard to eradicate, and seeds sown by the experiences of a child in the dental chair are very apt to bring forth fruit that, in after life, shall be the source of much trouble when future dental operations are necessary. Consequently, I believe it to be politic to be as gentle and careful in our first and early experiences with children as it is possible to be. Nevertheless, the fact confronts us that we have a duty to perform; and the question confronts us as to the best way to perform it? In the filling of the deciduous teeth, much depends upon the age of the child and the length of time that is necessary to preserve those teeth. My experience tells me that in those teeth which it is necessary to retain in the mouth but a short time longer, where the arch has been well expanded, and made ready for the permanent dentures; where absorption of the roots has become nearly completed. Little need be done, and that little need not, of a necessity, be very perfectly done. The child should be as gently and carefully handled as circumstances will permit. *But*, when decay in the temporary teeth begins *early*—and it may be years, possibly, before the permanent teeth may be expected to take their place—then the situation is entirely changed; and to be of any great benefit, our work must be of a more permanent nature, and the question arises: *How shall we make it so?* How thorough shall we be expected to prepare our cavities, and with what *materials* shall we fill them? The deciduous teeth, as we all know, are not as fully calcified and mineral in their formation as those that are to follow, consequently much more readily yield to those agents that cause decay, and painfully respond to the touch of the excavator and the burr. Still the fact presents itself, that a certain amount of excavating and preparation is imperative, or our labors will be of little use. And here I would suggest the greatest care and gentleness on the part of the operator. Let thoroughness be tempered with kindness, gentleness and discretion. Children are generally easily influenced; talk to them kindly and soothingly during the time that they are in your chair; divert, as much as possible, their thoughts from the ordeal through which they are passing, and make your sittings *short.* But make your work as thorough as possible in preparation of cavities as circumstances will permit. The filling materials that may be used are many, and the operator should be governed in their selection by the case in hand. Gold I should never use, for two reasons: One, that it is unnecessarily expensive; and the other, that the time expended for its proper manipulation is very trying to your little patient and returns no adequate results. When the rubber dam can be readily used, use it; the following results will be more satisfactory. The various preparations of gutta-percha that are now in the market in many cases are all-sufficient, though some of them, in my hands, have proved valueless. The so-called "Hill's Stopping," that was in the market some years since, was, I think, the best of its kind that I have ever used. The oxyphosphates as now prepared for our use are admirably adapted to many cases. But, when I think that the case demands a filling of more permanency of character, I use amalgam, often first lining the walls of the cavity with either a thin solution of gutta-percha or of the oxyphosphate of zinc. Now, in this suggestion, I feel that I may meet with very strong opposition on the part of some. But that fact makes not the slightest difference with me. I am asked to discuss a certain portion of the paper just read before you, and though my remarks are brief, still they are such as are the results of my former experiences. There was a time, which you and I both remember, when the fact that a man admitted that he used amalgam in any form, or in any way, was enough to damn him. That time, I think, has passed away. Amalgam, if properly prepared and properly used, may be, and has been, of much permanent benefit to many in the past, and may be to many in the future. And in its use in children's teeth, where quickness or operation and freedom from pain is a desideratum, I think amalgam has its place; and I consider it more permanent and lasting in many cases than any other agents we can use. Now, let it be understood that I am speaking now more particularly of deciduous, or children's teeth; I am speaking of past experiences as an operator, and as to the results of such experiences. The question before the house as I understand it, is: *How best to bend our efforts* for the preservation of children's teeth? Deciduous in the first place, which shall have for its end and aim the preservation finally of the permanent. The thoughts expressed by the writer of the paper, as a whole, meet my hearty approbation, but to discuss it at length would be, it seems to me, an almost endless task.

DISCUSSED BY W. H. JACKSON, D.D.S.

At the opening of this section of Dr. Hoff's paper, he says: "It seems to me that it is high time more notice was given to the eruption of the teeth in a regular order and harmonious position."

To consider the opening statement on this subject we are forced to go back beyond the appearance of the tooth, and this takes us into the realm of life-force.

Life is that mysterious power which produces the phenomena that distinguish living organisms from inanimate things.

The laws which govern life-growth are little understood, consequently what will be the product of two different temperaments coming together can not be foretold. Yet, by bringing together persons with strong physical bodies the offspring is more liable to receive a strong physical body.

During the slavery period in the South masters recognized this truth and selected males of great physical strength that the offspring might be of a like nature, hence of greater value on the market. But, as far as my knowledge extends, there has been no scientific work done in reference to the develpment of the teeth and maxillæ. It might be possible to develop almost any characteristic in the human family, as has been done in lower orders, were it possible to govern the mating of the sexes on a truly scientific basis to produce the object desired.

Some good work has been done in this line, in several directions on the horse for racing, roadsters and draught, on the bos for milk and beef, on the dog for his varied qualities.

I hardly think the doctor intended to enter the subject of heredity, however, as it will be next to impossible to remedy the present evil in that line, as there is no way of inducing people to submit to a scientific body in selecting a wife or husband, so we will deal with the teeth as they make their appearance. Then, if they are out of the normal position it is for us to correct the abnormality, and, so far as possible, produce perfect harmony for use and beauty.

The temporary teeth are so seldom abnormal in position that it is almost wasted time to consider them, for should there be conditions which we might deem wise to correct, the manipulation would be practically the same as with the permanent teeth.

Conservation of energy is of the utmost importance to physical life. So the questions arise in our minds—

First: At what time or period of dentition can we, with the least disturbance to the physical system, correct an abnormality?

Second: At what time or period of dentition may be obtained the best results?

Third: Contra indications, etc.

Fourth: What method of manipulation shall we follow to accomplish the desired end?

I will confine my remarks principally to the first two of these questions, merely touching the third and fourth.

In considering the first question we find that the younger the patient the less resistant are the tissues that we have to deal with. The processes which retain the teeth in position have not become so densely ossified, and absorption takes place much more rapidly from the greater vascularity, thus lessening the intensity of the irritation, and diminishing the length of time required to obtain the desired correction.

Matured tissues have habits of form which are very persistent, and so much so are they that even though you may seriously mangle a part, it will frequently return to the original shape and usefulness, and unless a part be entirely destroyed, there will be this tendency to return, owing, undoubtedly, to the life-force that directs.

While in the growing or formative period the habit has not been established and we are able to establish the habit of a correction or a deformity, so that in later years there will be the tendency to return to the habit you have established.

Corrective surgery in other lines has taken cognizance of these conditions, and are, many times, able to correct grave faults and bring about harmonious positions of the part, thereby making it more useful to the patient.

I am greatly surprised that our profession has not been impressed with the need of immediate manipulation for the correction of any deformity of the mouth and associate parts as soon as they may become apparent.

As a rule, it requires a very minute amount of directive power, while the tooth is growing, to bring it to its proper position, and a very small amount of irritation is produced if the appliance is made and adjusted properly.

On the other hand, should you leave the correction till later in life, when the tissues are fully formed, growth has practically ceased, and the habits and characteristics of the parts fully determined, much irritation and nervous tension will be produced, and has to be continued a much longer time to obtain a desired result in position.

Two patients, sisters, both extremely nervous, of like temperament, one eleven, the other nineteen years of age, will illustrate this plainly.

The younger patient's teeth were moved into position, and she was discharged between six and seven weeks, not even requiring any appliance to retain her teeth in the corrected position, while with the older, it took over four months to obtain as good results in position, and she is still, after eighteen months, obliged to wear an appliance to prevent them from returning to their original position.

Were these the only cases having this result I would not cite them as an argument in this discussion, but I find that it is, to a more or less modified extent, the rule rather than the exception, and this deferment in operation leads to much of the dissatisfaction to the dentist as well as the patient in this field of our practice.

Many of my most successful results have been when the operation was performed before the teeth had attained their growth.

So soon as a tooth begins to erupt in an abnormal position an appliance should be adjusted to direct it to its proper position, and the farther growth of the root will tend to strengthen and retain the tooth in the acquired position.

During the growth of the teeth and maxilla the processes are in an active state of development and readily follow up the moving tooth or teeth making it or them more solid.

In moving the teeth through fully-formed processes there is a tendency to produce cicatricial tissue which has a tendency to contract and return to its old position, but in the active or developmental stage there is little tendency to the cicatricial and much toward the growth of normal tissue.

We may be able to guide and direct the development into more symmetrical forms or useful positions with comparatively little trouble, but when the formative or developing period is passed and the tissues have assumed their permanent form, it, many times, requires an almost endless amount of patience, as well as misery, to change it, and even when changed it is liable to return to its old position and become like "The man who returned and found his room swept and garnished. Then goeth he, and taketh to him seven other spirits more wicked than himself; and they enter in and dwell there. And the last state of that man is worse than the first."

An illustration in this line may be of interest: A lady, aged twenty-five, had worn for four years an appliance to hold in position a left superior lateral incisor. By an accident the appliance was left out a week, and the tooth returned to its old position, articulating back of the lower teeth. From wearing the appliance large cavities came in the corrected and adjoining teeth although the patient said the appliance was removed twice a day and the teeth thoroughly cleaned.

The effect on the system may be slight or serious.

First: According to the nature and extent of the operation.

Second: The health and physical condition of the patient.

Third: The extreme irritability of the nervous system.

Due cognizance of all these conditions should be taken that we may modify our manipulations or withhold them altogether, as the necessity of the case may require.

I find that recuperation is much more rapid in the young, and seldom leaves any depressing after-effects on the child, while with the adult, depressing conditions often follow, and, it is well to bear this in mind, for though we may be able to correct a defect we might produce depressing effects from which the patient might not be able to recover.

The more simple an appliance that will accomplish the desired result, the better will the work be done. As a rule, appliances are too complex in mechanism for comfort and require too frequent manipulation to keep the forces operating, thus adding greatly to the discomfort of the patient, and oftentimes even forcing them to give up the operation.

Dr. Porter: There was a matter mentioned in Dr. Jackson's discussion which interested me very much, and which I would like to mention, and I would like something said about it more than I will say, and that is the correction of the teeth at different ages. There is no question, of course; and we all appreciate the fact that it is very much more easy to correct any irregularity early in life in children, and the sooner we can get at it the better. But there is one difficulty which I have met, and no doubt all of you have, and that is the fact that when deciduous teeth have been extracted too soon, where they have been decayed and extracted before they ought to have been, and before the other teeth have nearly erupted; for instance, in a case where the anterior part of the superior maxillary is very prominent, and the first molars—the deciduous molars—have been extracted, it seems to me there is a difficulty that we can not overcome. It is very difficult in that case, where the incisors are very irregular, to do very much with them, in my opinion, until after the bicuspids have been erupted. And in such cases I advise the patient to wait until the bicuspids have been erupted, until they are from twelve to fourteen years of age, and I can not see how we can commence any operations before that time.

Dr. Taft: There was a remark dropped incidentally by Dr. Cleland which seemed to call for a suggestion further, and that is this, that—as I thought I stated two or three times—I was not aiming to cover the entire ground; I did not aim to give any special formulas; I was discussing principles; I regard that as more important than special formulas. It is a very important matter that we should understand the principles upon which our practice is to be based, and from which it will grow, if we understand the principles well.

I fully concur in what he said in regard to the use of foods of certain kinds—all very good; everything he said was true and to the point; it was specific rather than general—had reference to particular uses rather than to principles, both of which is necessary. There seems to be no special diversity of opinion in regard to questions that were contained in the paper. The paper is happily expressed in that respect, and every branch and every line ought to have its due consideration, but I was requested to confine myself to a particular line of thought and I only endeavored to do that as best I could. Other parts of the paper, of course, have been well discussed and were exceedingly interesting.

Dr. Hall, of Ann Arbor: In regard to special treatment, I would like to call attention to the valuable use of aid to be derived from the use of nitrate of silver. Dr. Field spoke of filling with different materials, and he also spoke of the great necessity of saving the younger children's teeth until they could be removed by natural processes. With very young patients we have the most difficulty, even in handling them; and their teeth are small, and the gums cover the teeth so fully that we can not put a rubber dam on and treat them properly, and I would like to call the attention of the members to the use of nitrate of silver—a good many know all about it now, some, perhaps, do not—covering the cavities, the decayed portions, with nitrate of silver. The saliva is usually enough to dissolve the crystals a little, and they will find that the decay will cease at that point, and although it blackens the teeth for a time, it is much better to preserve them that much, even if we can not fill and contour them properly.

The meeting here adjourned.

